# Inter-strides variability affects internal foot tissue loadings during running

**DOI:** 10.1038/s41598-022-08177-1

**Published:** 2022-03-10

**Authors:** Coline Van Waerbeke, André Jacques, Eric Berton, Guillaume Rao

**Affiliations:** Aix Marseille Univ, CNRS, ISM, 163, avenue de Luminy, 13288 Marseille cedex 09, France

**Keywords:** Computational models, Musculoskeletal system, Biomedical engineering

## Abstract

Running overuse injuries result from an imbalance between repetitive loadings on the anatomical structures and their ability to adapt to these loadings. Unfortunately, the measure of these in-vivo loadings is not easily accessible. An optimal amount of movement variability is thought to decrease the running overuse injury risk, but the influence of movement variability on local tissue loading is still not known. A 3D dynamic finite element foot model driven by extrinsic muscle forces was developed to estimate the stress undergone by the different internal foot structures during the stance phase. The boundary conditions of different trials with similar running speed were used as input. Variability in bone stress (10%) and cartilage pressure (16%) can be expected while keeping the overall running speed constant. Bone and cartilage stress were mainly influenced by the muscle force profiles rather than by ground reaction force. These findings suggest, first, that the analysis of a single trial only is not representative of the internal tissue loadings distribution in the foot and second, that muscle forces must be considered when estimating bone and cartilage loadings at the foot level. This model could be applied to an optimal clinical management of the overuse injury.

## Introduction

The foot is a complex three-dimensional structure where numerous bones, ligaments, tendons, soft tissues, and muscles are interacting to provide stability, cushioning, and propulsion to the rest of the body. As the sole contact point with the ground, the foot is subject to repetitive strong mechanical constraints during dynamic tasks, increasing the risks of developing acute or overuse injuries, such as plantar fasciitis, stress fracture^1^, bedsores^2^, and osteochondral lesions^3,4^. These overuse injuries could result from abnormal tissue loading^5,6^ and more precisely from an imbalance between the loading and the regeneration capacities of the tissue^7^. Thereby, it is of great interest for clinicians, to quantify the strain and stress undergone by the different anatomical structures while the body is moving.

The magnitude of the local tissue loading depends on several extrinsic and intrinsic factors, with the most important ones being the external forces, the geometry and mechanical properties of the anatomical structures, the intersegmental loads, the initial velocities and positions, and the forces produced by the muscles acting on the joints. Direct measurements of those loads are not possible in vivo*;* therefore, the use of biomechanical modeling is necessary. From a computational point of view, providing a biomechanical model able to estimate the tissue loading while considering all the environmental characteristics (i.e., external forces, geometrical and mechanical characteristics of the tissues, muscle forces, etc.) is highly challenging because of their different natures. Indeed, muscle forces that set the body in motion are usually assessed using (neuro)-musculoskeletal models of the body^8–12^, whereas tissue loadings are estimated using a finite element (FE) analysis of the concerned body structures^13,14^.

Previous studies using FE modeling of the foot have provided interesting results while being only static, quasi-static, or based on a two-dimensional representation^13,15–21^. For instance, Qian et al.^21^ developed a 2D FE model of the foot able to predict with a good agreement the ground reaction forces, the center of pressure as well as the plantar pressure. However, 2D models may not accurately represent the very complex 3D structure of the foot and its inner interactions during the gait. Indeed, analyzing the stress and strains using 2D models is very unlikely to capture the precise behavior of each structure forming the foot, mainly due to the absence of symmetry within this anatomical structure. Extrapolating an appropriate clinical treatment based on results provided by an (over-)simplified model of the foot can be questionable^21,22^. More complex and realistic FE models are needed and have been developed by coupling both a musculoskeletal model and a 3-D FE model for the joint of interest. Besier et al.^23^ created a knee joint FE model driven by the estimated forces derived from an EMG-driven musculoskeletal model. This model, validated with experimental data of the patellofemoral joint contact area and patellar orientation, was able to estimate the cartilage stress in the patellofemoral joint and compare the values on the different cartilages around the joint.

Another study from Scarton et al.^17^ developed a 3D foot FE model designed to help prevent diabetic foot by estimating the foot internal stresses. Both studies used quasi-static simulations and therefore focus on precise instants of the gait cycle and do not consider the overall movement and energy flow. Indeed, using a dynamic simulation, Qian et al.^21^ showed a 10 to 30% improvement in accuracy of the peak plantar pressure estimation compared to a quasi-static simulation. Recently, a 3D dynamic FE model of the foot driven by muscle forces and the ankle joint reaction force was developed by Chen et al.^24^ using a similar framework. The main strength of this study is to combine a 3D FE model of the foot and a dynamic simulation which increases the accuracy of the predictions. But this model might underestimate the influence of the overall movement of the body on the foot while considering only the dynamics of the foot–ankle complex.

A movement is never repeated exactly the same way even during a cycle task such as locomotion^25^. This intra-individual variability between strides is functional and could lead to different loadings of the anatomical structures resulting in wider stress distribution and consequently reduced local loadings over time^26^. An alteration of the gait variability could lead to or be the consequences of an injury or a pathology such as patellofemoral pain syndrome, Parkinson’s, and Huntington's diseases^27,28^. For that reason, the gait variability needs to be considered when analyzing local tissue loading since one trial might not be representative of a participant’s gait and thus of the local tissue loadings. Currently, the influence of movement variability on local tissue loading is not known and might hinder an optimal clinical management of the injury.

The aim of the present study is to explore the effects of intra-individual gait variability on the repetitive internal tissue loadings in the foot during running. Experimental data acquisition and musculoskeletal modeling were used to estimate muscle forces for different trials, and these forces and boundary conditions were further used to drive a 3D dynamic FE model of the foot, simulating running tasks. The objective is thus to adequately account for the inherent locomotion variability within the model and further to explore its consequence on the local tissue loadings.

## Methods

### FE model of the foot

**Figure 1:**
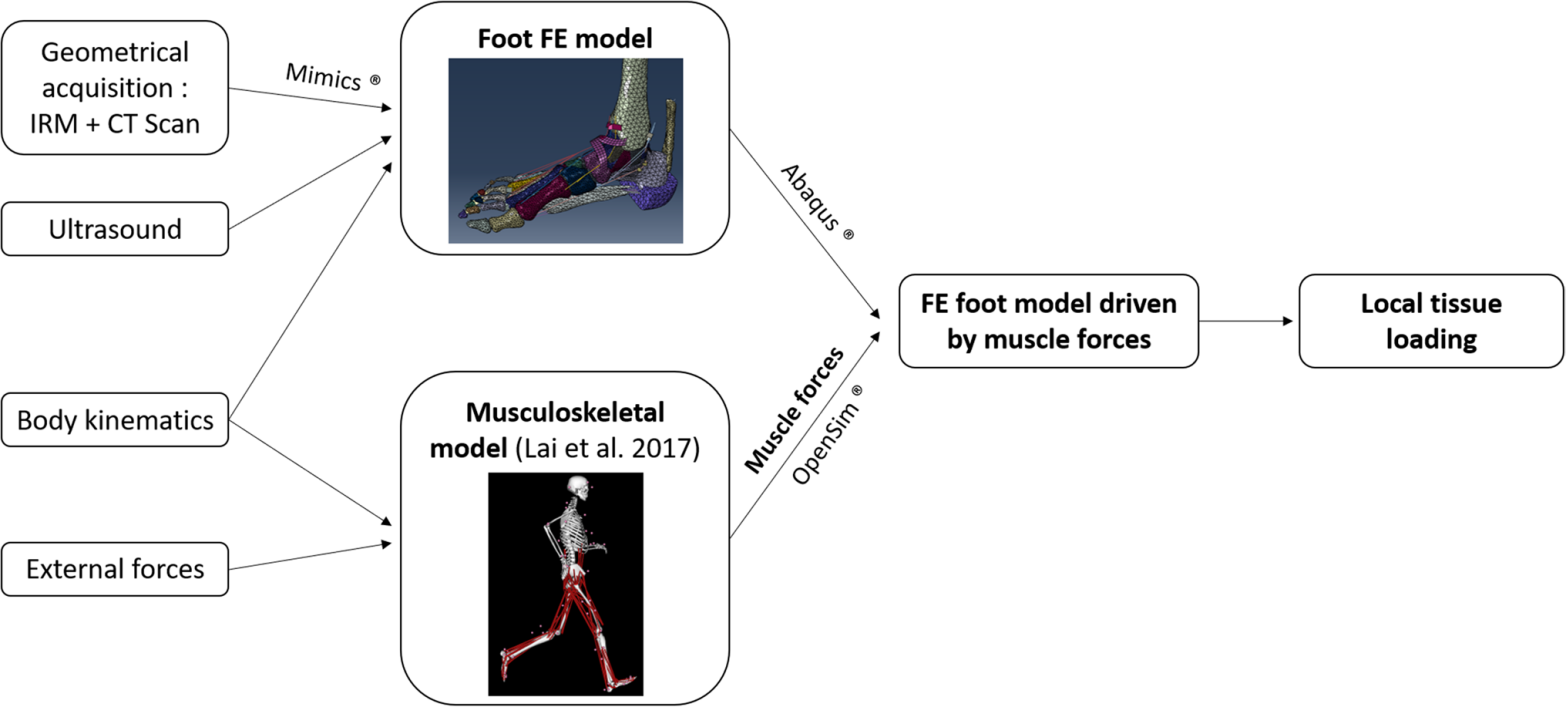
Complete workflow of model building, including the FE part and the tendon forces estimations.

**Figure 2:**
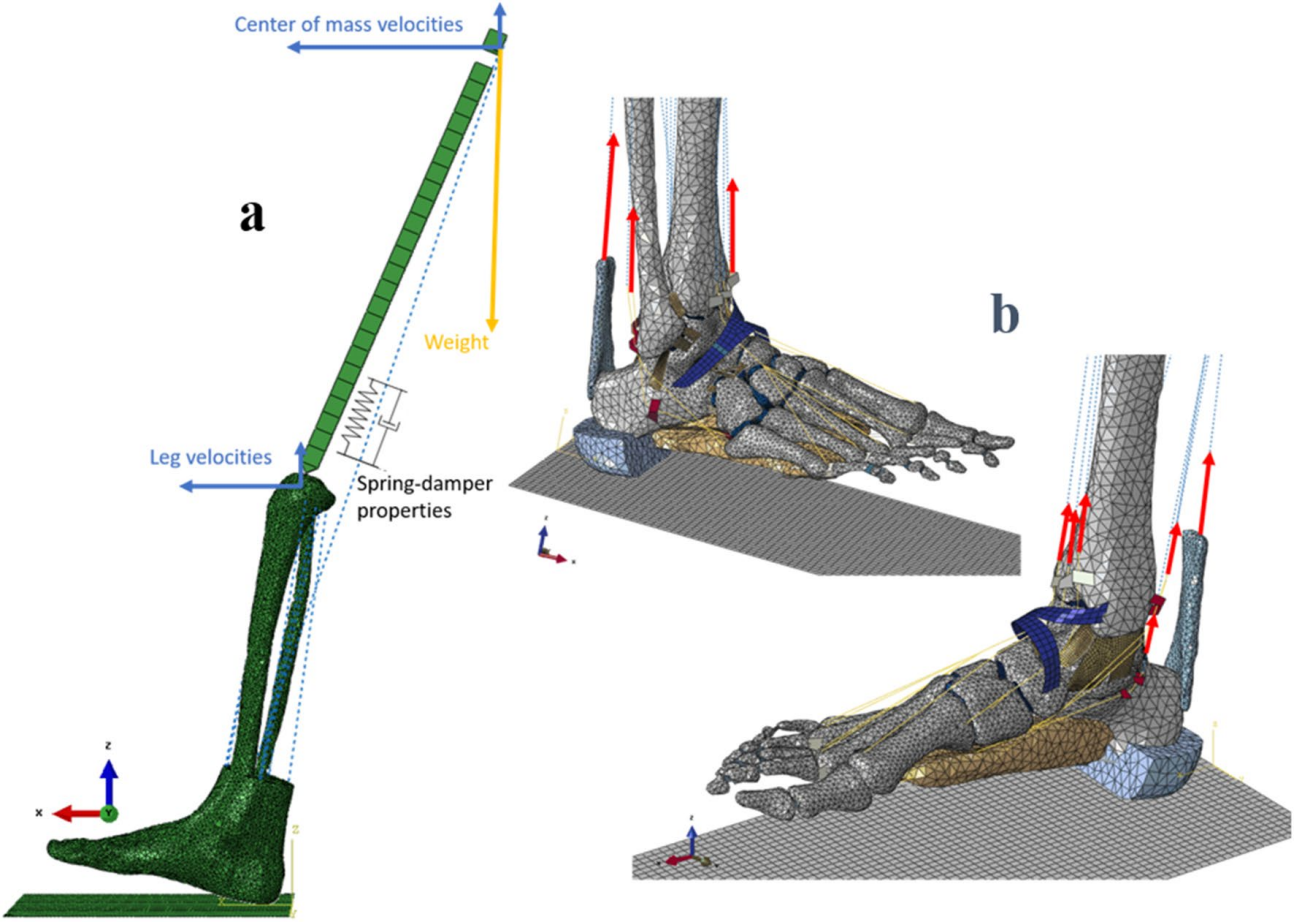
(**a**) FE model of the foot, including a mass-spring-damper system, the initial antero-posterior and vertical velocities (in blue) and the weight applied at the center of mass (in yellow). (**b**) The model is actuated by applying an experimentally-derived force on the tendons of the 9 extrinsic foot muscles (in red).

A framework of the different steps involved in the development of the model is presented in Fig. 1. The 3D FE model of the foot, actuated by nine extrinsic foot muscles, includes geometrical acquisition for the foot–ankle complex and the tibia. To simulate the Head-Arm-Trunk complex (not included in the FE model) and its influence on the foot loading, a simple representation of the femur and a 70.8 kg mass-equivalent element located approximately at the subject center of mass were added. A mass-spring-damper system connects the virtual center of mass to the bottom part of the tibia (see Fig. 2a).

The model was developed based on medical imaging data of a single adult male subject (30 years, 1.80 m, 72 kg). The participant was a frequent runner (20 km/week) and a rearfoot striker. All methods were carried out in accordance with the Declaration of Helsinki and all the procedures have been approved by the Aix-Marseille University ethics committee. Informed consent was obtained from the participant. MRI and CT scan data were obtained from this subject with the knee joint fully extended and the ankle joint in a neutral position. For each acquisition, the ankle joint was kept in neutral position by using an anatomical cast of the lower limb. CT scans were further imported in Mimics^©^ software to extract the geometries of the bones. MRI data were then used to determine the paths of the tendons, ligaments, or fascia (more details in the model construction in Supplementary materials).

The complete model results in 49 114 nodes and 221 398 elements. In order to drive the model using experimentally-derived muscle forces, 9 tendons were included in the FE foot model (see Fig. 2b).

Mechanical properties of tissues were either taken from the literature as direct expressions of mechanical models (for example Young’s modulus or Poisson’s ratio for linear models) or were extracted from stress–strain literature relationships, using established formulations of potentials, such as Ogden or polynomial representations (see Supplementary materials). For the Achilles tendon and the heel pad, specific studies including ultrasound analysis for the first structure (see Fig. 1) were conducted, to measure the potentials strain stress relationship (see Supplementary materials)^29^.

### Muscles forces estimations

Experimental data were acquired during three representative trials where the subject was asked to run 40 m in the motion capture set up at a preferred speed (3.6 m/s). The participant was instructed not to adjust his stride length to target the force plate and watch in front of him. A trial was accepted if the right foot impacted the middle of the force plate and the mean trial velocity was within a ± 5% limit compared to the preferred speed. Trials were performed barefoot to avoid the shoe influence. The participant was equipped with 71 infrared reflective markers. Using conventional motion capture setup with 19 cameras and one force plate, kinematics of the lower limb (Qualisys AB, Sweden) and ground reaction forces (Kistler, Switzerland) were recorded synchronously. The sampling frequency was respectively set at 200 Hz and 2000 Hz. A new calibration step has been added with the Cal Tester (C-Motion Research Biomechanics, USA) to improve the accuracy of the center of pressure position by spatially synchronize the force platform in the field of the cameras. Kinematics and kinetics data were filtered using a fourth-order Butterworth filter with a cut-off frequency of 10 Hz.

The musculoskeletal model used is based on the work of Lai et al.^10^. The maximum isometric forces of all muscles were multiplied by two to adjust the participant’s strength and to avoid reaching a plateau during the estimation^30^. The Static Optimization tool from OpenSim^8^ was used to estimate the nine extrinsic foot muscle forces based on experimental data (triceps surae, tibialis anterior, tibialis posterior, extensor hallucis, extensor digitorum, flexor hallucis, flexor digitorum, peroneus brevis, peroneus longus).

### Boundary conditions

The boundary conditions of the FE model consisted of the experimentally measured initial 2D (anteroposterior and vertical) velocities at touchdown of the foot and the center of mass element, as well as the estimated time dependent muscles forces acting on the foot. The apparent weight of the model was applied as an external vertical force, on the element situated at the center of mass, constant for a running simulation (see Fig. 2a).

### Simulation

The explicit version of the FE solver Abaqus^©^ (6.14, Dassault Systems) was used to simulate the impact and stance phase of the gait cycle. Once the boundary conditions were set, the model was driven by time dependent muscle forces and ground contact only (see Fig. 2).

### Validation and output data

To validate this FE foot model, predicted anteroposterior and vertical ground reaction forces and experimental data of the force plate were compared for the three trials. A comparison of the time-evolution of the angle between the foot sole and the ground was also conducted between results from numerical simulation and kinematics data. This angle was computed in the sagittal plane using the coordinates of both the heel and the second metatarsal head markers (nodes in the computation).

Output data focused on anatomical structures often affected by running injuries. It consisted in the time-evolution of the contact pressure on the tibiotalar cartilage as well as the stress undergone by the second metatarsal for all trials during the stance phase. Von Mises constraints as well as the components of the stress tensor were reported at the integration point.

## Results

### Experimental data and muscle forces

**Figure 3:**
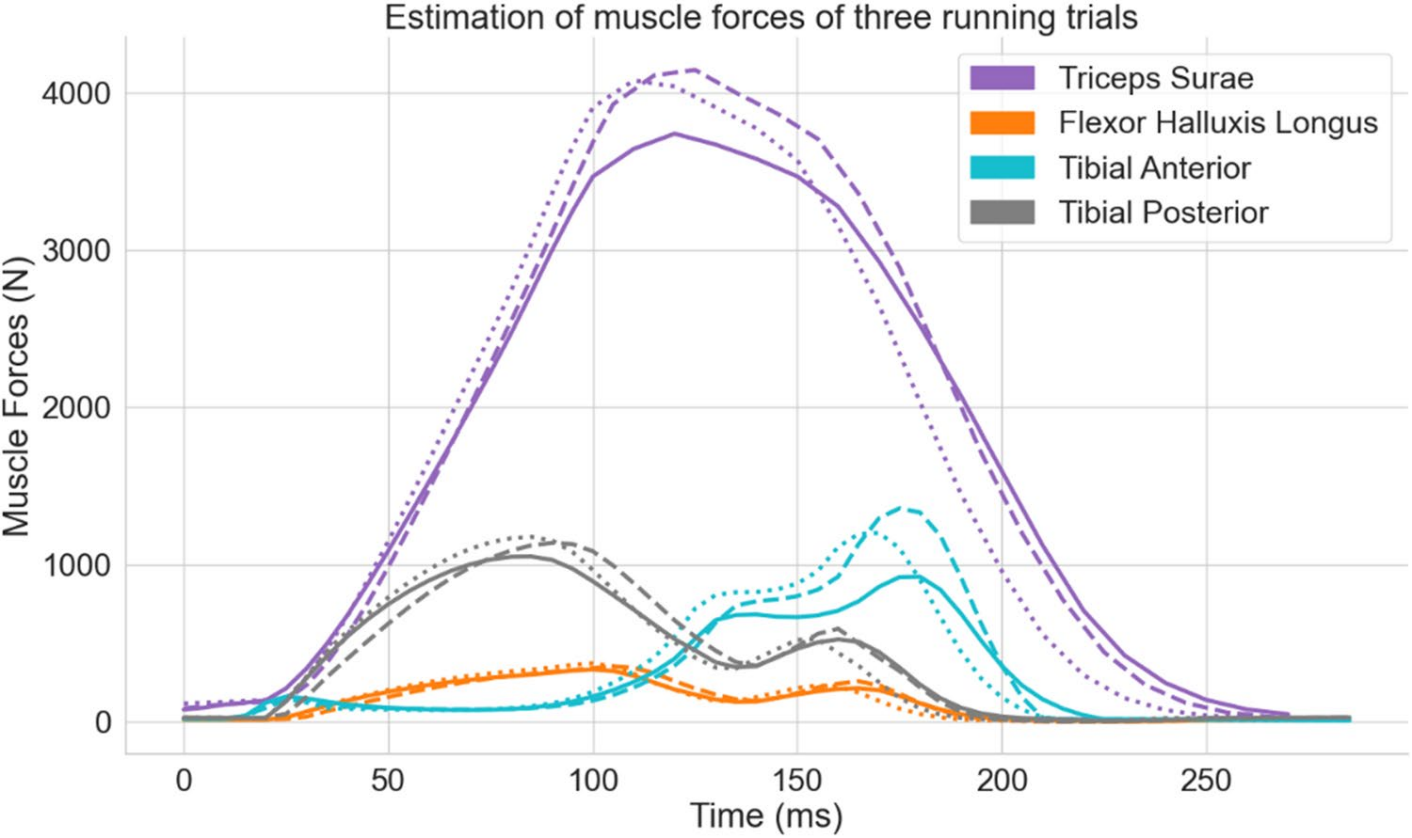
Time dependent forces acting on the four most important tendons involved in the foot movement (triceps surae in purple, flexor halluxis in orange, tibial anterior in cyan, and tibial posterior in grey), during three non-consecutive running stance phases. Each trial is represented by a different line style (1 = solid, 2 = dashed & 3 = dotted). The five other muscles are not displayed due to a smaller developed force compared to the previous four.

**Figure 4:**
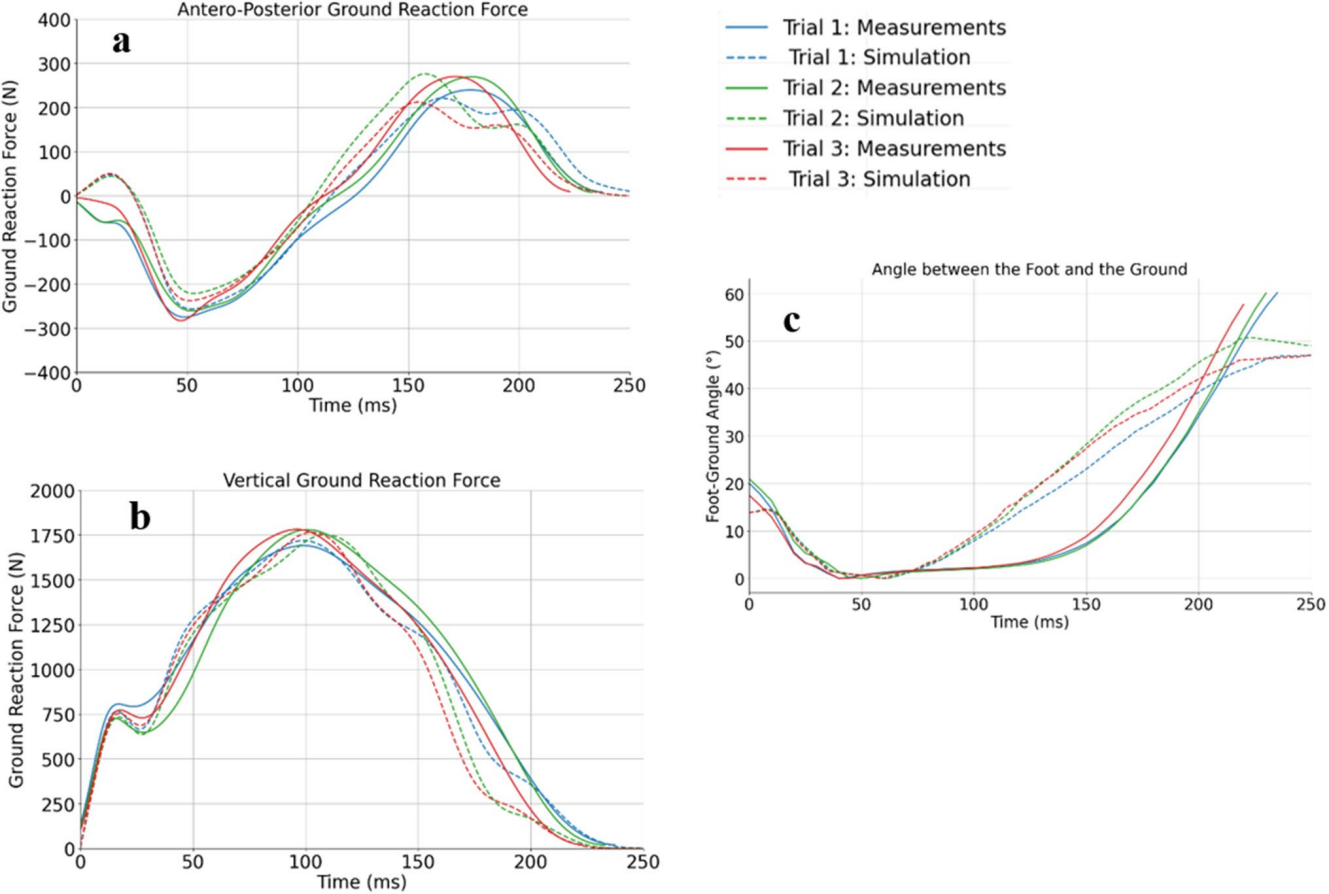
Results of model validation during the stance phase. Comparison between the computed (dotted line) and measured (solid line) (**a**) antero-posterior ground reaction forces, (**b**) vertical ground reaction forces and (**c**) angle between the foot and the ground of three running cycles (trial one in blue, trial two in green et trial three in red).

The results of three running trials are reported. The running speed of each trial was respectively of 3.5 m/s, 3.67 m/s, and 3.66 m/s, and stood within a 5% variance of the preferred speed. Initial velocity conditions for the foot and the center of mass at touchdown are reported in Table 1. Predicted muscle forces were compared to EMG data extracted from literature and/or to the results of other musculoskeletal simulations studies, to ensure their validity^31–34^. Fig. 3 shows the estimated forces of the four major muscles acting on the foot during the stance phase for the three trials. Notable inter-trials differences are found in the force amplitude for both the triceps surae and the tibial anterior. Smaller muscle forces were estimated during the slowest trial (3.5 m/s), compared to the fastest one (3.67 m/s), with a force maximum decrease of respectively 10% and 37% for the triceps surae and the tibial anterior (see Fig. 3). Lower differences were found in the vertical ground reaction peak with the lowest trial showing only a 5% decreased force (1691 N) compared to the two other trials (1780 N and 1781 N) (see Fig. 4b, solid lines).

**Table 1:** Initial velocity conditions for the three running cycles.

	Center of mass		Foot–ankle complex	
Initial velocity (m/s)	Antero-posterior	Vertical	Antero-posterior	Vertical
Trial no. 1	3.54	− 0.56	1.05	− 1.20
Trial no. 2	3.73	− 0.47	1.17	− 1.12
Trial no. 3	3.71	− 0.49	1.00	− 1.18

### Model validation

Each simulation lasted between four to six hours on a Dell Optiplex 9020 computer (Dell, USA). A mean difference of 1.9% is observed between experimental (respectively 0.238 s, 0.234 s, and 0.224 s) and computed contact times (0.236 s, 0.224 s, and 0.222 s). Despite a slight delay during the last phase of stance, the similarity is large between experimental and computed curves for both horizontal and vertical ground reaction forces. (see Fig. 4a,b). Experimental data shows that the foot stays flat for approximately 75 ms during the stance phase while in the computed data, this angle increases just after reaching the minimum with the foot sole remaining completely on the ground for less than 30 ms (see Fig. 4c). Despite these discrepancies, the overall amplitudes and initial behavior after contact were in close agreement.

#### Bone and cartilage stress

The three trials showed differences in the amplitude and timing of the peak average Von Mises stress which occurs respectively at 120 ms, 120 ms, and 110 ms. The highest peak average value (15.7 MPa) occurs during the second trial and is respectively 10% and 4.6% higher compared to the slowest and the third trial (14.2 MPa and 15 MPa).

**Figure 5:**
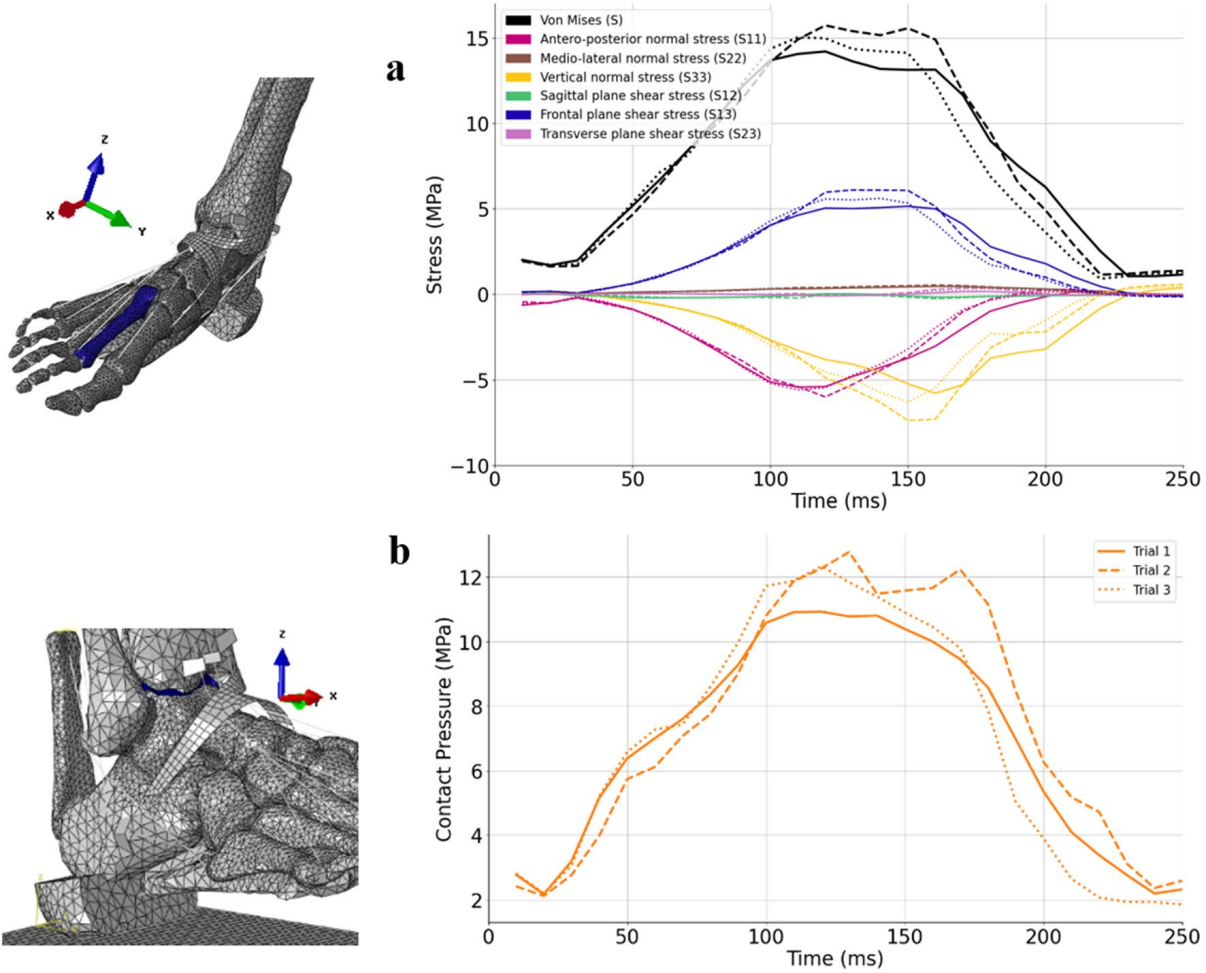
(**a**) Average of Von Mises stresses (in black) and of the stress tensor components (in color) on the second metatarsal for the three trials during the stance phase. S11(magenta), S22 (brown), and S33 (yellow) are respectively the normal stresses in anteroposterior (x-axis), mediolateral (y-axis), and vertical (z-axis) directions. S12 (green), S13 (blue), and S23 (purple) are respectively the shear stresses in sagittal, frontal, and transverse planes. (**b**) Average of the contact pressure undergone by the tibiotalar cartilage. For both graphs, each trial is represented by a different line style (1 = solid, 2 = dashed & 3 = dotted).

Antero-posterior (x-axis) and vertical (z-axis) compressive (normal) stresses, as well as shear stress in the transverse plane (in the x-axis direction and in the perpendicular plane to the z-axis), account for the majority of Von Mises stress in the second metatarsal bone (see Fig. 5a). However, the distribution of the stress tensor components is different depending on the trials. For instance, despite having the lowest average Von Mises stress during the stance phase compared to the other trials, the first trial shows a higher anteroposterior compressive stress.

Similar to the Von Mises stress on the second metatarsal, a difference is observed in the timing and amplitude of the average contact pressure peak of the three running trials (see Fig. 5b). The maximum average contact pressure occurs at respectively, 120 ms, 130 ms, and 120 ms. The highest peak average value (12.8 MPa) occurs during the second trial and is respectively 16% and 4% higher compared to the first and the third trial (10.9 MPa and 12.3 MPa).

For all trials, the highest average Von Mises Stress on the second metatarsal and the highest average contact pressure on the tibiotalar cartilage occur slightly (20 ms) after the maximum vertical ground reaction force and approximately at the time when the triceps surae force reaches its maximum amplitude.

**Figure 6:**
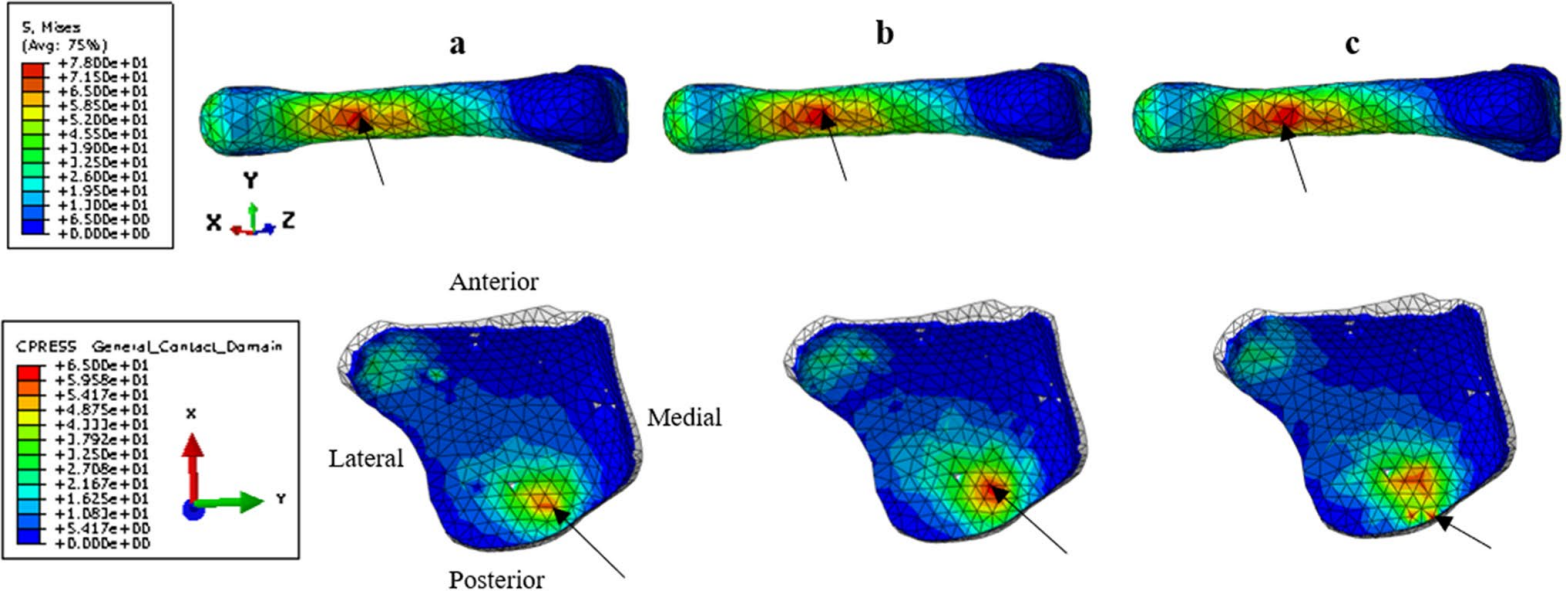
Example of field data that can be extracted from the model. In the first row, the distribution of Von Mises stresses on the second metatarsal at the time of the peak of the average Von Mises stresses on the whole bone for the three running trials (t = 120 ms for trials 1 and 2 and t = 110 ms for trial 3, respectively **a**–**c**), view from below. In the second row, the distribution of the contact pressure at the time of the peak of the average contact pressure on the tibiotalar cartilage for the three running trials (t = 120 ms for trials 1 and 3 and t = 130 ms for trial 3). Black arrows represent the location of the maximum Von Mises stress or contact pressure.

Figure 6 shows the estimated Von Mises distribution on the second metatarsal for all trials at the time of the peak average Von Mises stress on the whole bone as well as the estimated contact pressure distribution on the tibiotalar cartilage at the time of the peak average contact pressure. Higher internal stress on the second metatarsal is found on the plantar surface of the neck and distal shaft. A larger surface of high stresses (> 65 MPa) is found on the second and third trials compared to the first one (see Fig. 6). Similar observations are made for the contact pressure on the tibiotalar cartilage with a larger surface of high pressure for trials 2 and 3.

## Discussion

Most overuse injuries are due to repetitive abnormal loadings on anatomical structures^5,6^. Running gait cycles are not identical and the influence of the inter-stride variability on the loadings of the anatomical structures is unknown. Analyzing the stress distribution on the structure in question for a unique trial does not consider this intra-individual variability occurring across strides and therefore limit the clinical applications of the obtained results. The purpose of this study was to investigate the effects of the inherent variability observed during locomotion on repetitive internal tissue loadings during running. A dynamic 3D FE Model of the foot driven by experimentally derived muscle forces was developed to predict internal stresses of foot structures that are otherwise non-measurable.

This model was validated by comparing the simulated spatio-temporal, kinetic and kinematic data with experimental data. Ground contact times were similar between the experimental and simulated data with an average difference of less than 2%. The kinematics of the foot angle with the ground showed a similar trend despite some deviations observed during late-stance (discussed further in the limits). Akrami et al.^35^ showed the importance of considering both the vertical and anteroposterior reaction forces during simulation so that the braking and propulsion phases are consistent with reality. Despite a slight delay during the propulsion phase, our results showed a strong agreement between the profiles of the simulated ground reaction forces and those obtained with the force plate, thus giving credit to the data issued from the simulations.

Most of the models already presented^21,24,35,36^ represent only the foot–ankle complex. Yet, running dynamics is partially explained by the stiffness and damping properties of the lower limb, thus impacting the loading of the foot^37^. Considering these properties is essential to obtain a model close to reality. The main strength of the model is the single-case design. Indeed, given the accuracy of the data we are analyzing, the choice was to create a subject-specific finite element model of the foot since a simplification of the model could hide the expected results. In addition, studies have been specifically performed to best represent the material properties of the heel pad and Achilles tendon^29^. Experimentally derived muscle forces were also acquired and computed for the same participant in order to match as closely as possible all the input data. The accuracy of the spatio-temporal, kinetic and kinematic data obtained through the FE model ascertains that this model can reliably reproduce the biomechanical responses of the foot during the stance phase of running.

Three simulations were run with the initial conditions and estimated muscle forces of three different running trials to observe the influence of a modification in these boundaries and loading conditions on the stresses undergone by the second metatarsal and the tibiotalar cartilage. The differences in the boundary and loading conditions of the three trials were small, yet significant (less than 6% for the initial velocities and up to 10% on average for muscle forces estimations) and lead to significant variability in the internal tissue loadings. Indeed, a large difference in the peak tibial anterior force (37%) is observed when comparing two trials having only a 0.17 m/s speed difference. This variation could be explained by the noticeable difference observed in the slope and amplitude of the Achille tendon force leading to changes in the co-contraction and therefore affecting the tibial anterior force. Results showed consistency between inter-trial differences in the stress undergone by the second metatarsal and the tibiotalar cartilage with the differences in the initial and loading conditions of the model. Interestingly, the inter-trial differences in amplitude and timing between the curves representing the average Von Mises stress on the bone and the contact pressure on the cartilage are similar to the differences observed on the vertical ground reaction curves and even more with the force of the triceps surae. For instance, a 10% decrease in the estimated maximal force by the Achilles tendon also reduced the max average Von Mises stress by about 10% and the contact pressure by 16%. However, the findings indicated that the distribution of the stress tensor components was not proportional to the overall Von Mises stress. Indeed, the trial presenting the highest anteroposterior compressive stress had the lowest average Von Mises stress. Those results could be explained by the variability in the foot orientation at touchdown which influences the internal loading^24^. Consequently, when analyzing the Von Mises stress, attention should be given to the distribution among the stress tensor components. The results also showed lower stress undergone by the second metatarsal bone and by the cartilage during the slowest trial, which also had lower muscle forces amplitudes.

Of note, all the analyzed trials were within the common ± 5% acceptance limit for experimental running speed. Thus, significant variability in bone stress (10%) and cartilage contact pressure (16%) can be expected while keeping the overall running speed within the accepted variability (5%). Taken together, these observations prove that the model is sensitive and accurate enough to analyze the effects of the variability observed in the initial conditions on the repetition and distribution of internal stresses in the foot during running.

The maximum stresses reported on the second metatarsal were located at the neck and on the distal plantar part of the bone. Our results are in agreement with the literature since according to Chuckpaiwong et al.^38^, approximately 90% of second metatarsal stress fractures occur in the distal part of the bone, generally along the metatarsal shaft or the neck. The maximum Von Mises stresses estimated on the second metatarsal were between 73 and 77 MPa. These values are approximately 40% higher than the average maximum stresses estimated in the study of Ellison et al.^39^, which aim was to create a subject-specific model of the second metatarsal only to quantify the stress undergone on this bone. However, in Ellison’s study as opposed to the present one, the simulations did not include the muscle forces, were quasi-static, and performed at only 3 instants including the instant of peak vertical ground reaction force. In our results, the maximum stress peak is slightly shifted in time with respect to the vertical force peak and closer to the peak in muscle forces. Considering both the internal (muscle forces) and external (ground reaction forces) loadings is thus of paramount importance when computing internal stress. Our results also reinforce the interest in using a dynamic simulation allowing us to get data on the entire stance phase and not only on a limited number of instants. From a clinical perspective, the reported 3D finite elements foot model driven by muscle forces is suitable to estimate the location and intensities of mechanical loads while considering the majority of the variables that can affect these loads (anatomical structures geometries and properties, movement-related boundary conditions, muscle forces).

A model always has limitations since it is a mathematical representation of reality. In this study, the strength is also the weakness since the main limitation is the difficulty to generalize the results due to the single-case design. The second limitation is related to the discrepancy observed between the simulated and experimental angle between the foot sole and the ground. This difference was considered in our analysis and the focus was on the mean stress and pressure peak occurring at around 100–120 ms, before the largest divergence. The small difference still observed at those instants could be explained by the differences in the angle computations (using real markers on the skin for the experimental angle and nodes on the bones for simulated angle). In the future, the use of a multi-segment musculoskeletal foot model could potentially also help in this regard. However, adding more complexity to the initial musculoskeletal model could generate more errors and was thus not chosen in this first step. The third limitation concerns the estimates of the muscular forces, calculated with the static optimization. This tool uses the motion of the model described by the positions, velocities, and accelerations to solve the equations involving the joint torques and the muscle forces, but it does not consider the muscular activity. One of the perspectives would be to integrate EMG data to guide the static optimization and obtain estimates of muscle forces closer to the biological reality, especially for a population with altered muscle activation patterns. Currently, bones are modeled as a homogeneous material. To improve the prediction of stresses within the bone, future work will focus on creating a heterogeneous bone considering the trabecular and cortical parts.

Only extrinsic foot muscle forces were estimated and included in the model. Indeed, adding the intrinsic muscles is a complex task and could lead to some uncertainties since information regarding their activation is lacking. Recent studies highlight their active role in the foot windlass mechanism and their importance in tasks where the foot stiffness varies a lot (uneven ground or change of speed for instance)^40,41^. They were kept aside for the first version of the model.

The novelty of this fully personalized multiscale foot model is its ability to account for variability in model inputs to improve the estimation of local internal stress. The workflow of our study could therefore be extended and applied to an injured population to get more insights in the link between variability and overuse injuries, like stress fractures.

## Conclusion

A 3D dynamic FE foot model was developed to investigate the effects of the boundary and loading conditions variability, as observed in multiple running trials, on the foot internal tissue stresses distribution. The variability observed in bone and cartilage stresses is consistent with the variability measured with experimental data, yet also showed differences related to the combined implementation of foot anatomical structures geometries and properties with experimentally derived muscle forces. Therefore, this model is a useful tool and could further be applied to understand the abnormal loadings leading to stress fracture injury by focusing on the stress variability (insufficient or excessive). The application of the model could also be extended to other foot-related injuries.

## Supplementary Information

Supplementary Information.
