# Supplementary material for: Inter-strides variability affects internal foot tissue loadings during running
Source: Sci Rep. 2022 Mar 10;12:4227. doi: 10.1038/s41598-022-08177-1 (PMC8913624; doi:10.1038/s41598-022-08177-1)
Supplement: Supplementary file 1 — Supplementary Information. [file 41598_2022_8177_MOESM1_ESM.pdf]

Inter-strides variability affects internal foot tissue loadings during running

Authors information :

Coline Van Waerbeke[1]*, André Jacques[1], Eric Berton[1], Guillaume Rao[1]

[1]Aix-Marseille Univ, CNRS, ISM, Marseille, France

e-mail: coline.van-waerbeke@univ-amu.fr

More details :

Coline Van Waerbeke*

UMR 7287 « Institut des Sciences du Mouvement »

CNRS et Université Aix-Marseille,

163, avenue de Luminy, F-13288 Marseille cedex 09, France

e-mail: coline.van-waerbeke@univ-amu.fr

André Jacques

UMR 7287 « Institut des Sciences du Mouvement »

CNRS et Université Aix-Marseille,

163, avenue de Luminy, F-13288 Marseille cedex 09, France

e-mail: andre.jacques@univ-amu.fr

Eric Berton

UMR 7287 « Institut des Sciences du Mouvement »

CNRS et Université Aix-Marseille,

163, avenue de Luminy, F-13288 Marseille cedex 09, France

e-mail: eric.berton@univ-amu.fr

Guillaume Rao

UMR 7287 « Institut des Sciences du Mouvement »

CNRS et Université Aix-Marseille,

163, avenue de Luminy, F-13288 Marseille cedex 09, France

e-mail: guillaume.rao@univ-amu.fr

Supplementary Materials

Supplementary material n°1: *Model construction.*

The geometrical datasets were further meshed through several phases. The first main step was to mesh in tetrahedron elements the bones structures and the surrounding soft tissues representing heel pad, intrinsic muscles, fat volumes, skin, etc. The mesh was separated into parts corresponding to the bones (28) and three separate soft tissue volume zones: the heel pad, the intrinsic muscles situated between the plantar fascia and the bottom of the bone structure, and the main foot soft tissues (see Fig a). These parts were directly coupled by common nodes of the mesh. The second step was to mesh the cartilage volumes, define both by the nodes surfaces and limiting surfaces extracted by general anatomic descriptions and MRI data. The cartilages were meshed using tetrahedron elements, generated directly on the volumes with non-structured meshing techniques, or by a split of thin prism volumes. The third step was the meshing of the ligaments, where a direct surface based quad mesh has been generated. The last step was the generation of the tendons and tendon's pulleys that have been created directly on the FE model editor (Abaqus CAE) using positions from CT Scan, MRI, and theoretical anatomic drawings (see Fig. b). The pulleys were represented as surface quad elements, the tendons by unidirectional elements, for all the involved tendons (n=8), but not for the Achilles tendon, meshed with a volume tetrahedral set of elements.

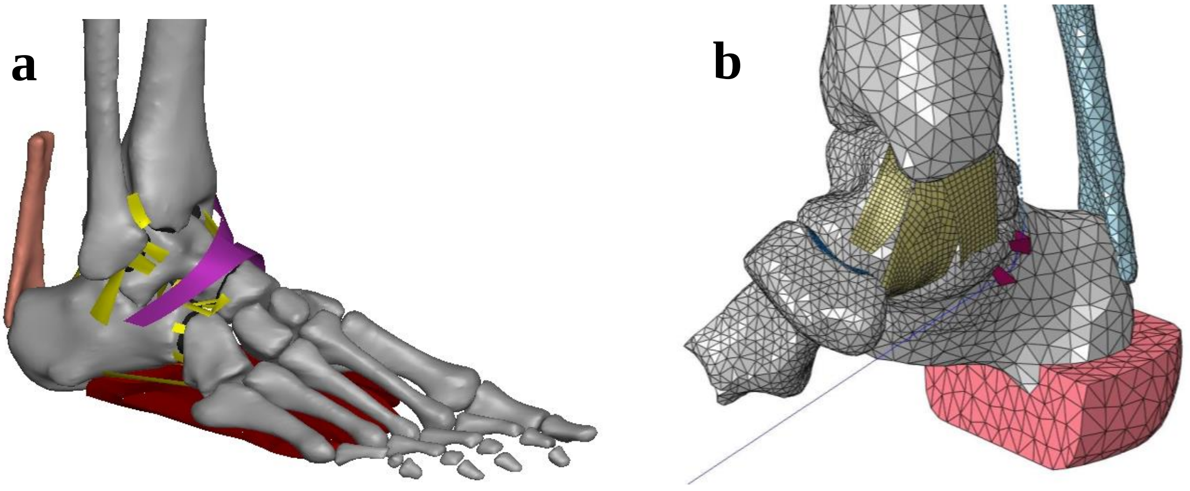

Figure in supplementary material n°1

Supplementary material n°2: *Material properties and element type of the foot FE model.*

Component	Materials Properties	Density	Values	Elements type	Cross-sectional areas and thickness of shells
Bones (MEL)[13]	Linear Elastic	1800 kg/m^3	E = 7300 MPa<br>$\nu = 0.3$	3D-Tetrahedron	
Cartilages	Hyperelastic (third order Ogden)	1500 kg/m^3	$\mu_1 = -4527$, $\alpha 1 = 4.98$<br>$\mu_2 = 2228$, $\alpha_2 = 5.43$<br>$\mu_3 = 2300$, $\alpha_3 = 4.55$<br>D1 = D2 = D3 = 0	3D-Tetrahedron	
Ligaments (SSEL) [35–38]	Hyperelastic (third order Ogden)	1500 kg/m^3	$\mu_1 = -9665$, $\alpha_1 = 24.5$<br>$\mu_2 = 5253$, $\alpha_2 = 25.0$<br>$\mu_3 = 4420$, $\alpha_3 = 23.9$<br>D1 = D2 = D3 = 0	2D-Quadrangles	1 mm
Plantar Fascia (MEL) [13]	Linear Elastic	1500 kg/m^3	E = 350 MPa<br>$\nu = 0.3$	2D- Triangles	3 mm
Main foot soft tissue (MEL)[13,39]	Hyperelastic (second order Ogden with damping)	1070 kg/m^3	$C_{10} = 0.17113$<br>$C_{01} = -0.11683$<br>$C_{20} = 0.078$<br>$C_{11} = -0.04638$<br>$C_{02} = 0.01702$<br>D1 = 1.822636, D2 = 0<br>$\alpha = 0.6$, $\beta = 0$	3D-Tetrahedron	
Heel Pad (SM)	Hyperelastic (first order Ogden)	1000 kg/m^3	$\mu_1 = 0.0453$, $\alpha_1 = 10.91$<br>D1 = 0	3D-Tetrahedron	
Tendon (MEL)[40,41]	Linear Elastic (with damping)	1500 kg/m^3	E = 7000 MPa<br>$\nu = 0.3$<br>$\alpha = 0.4$, $\beta = 0$	Linear elements (truss)	4 mm^2
Achilles Tendon (SM)[29]	Hyperelastic (third order Ogden)	1500 kg/m^3	$\mu_1 = -90049$, $\alpha_1 = 2.00$<br>$\mu_2 = 60735$, $\alpha_2 = 4.00$<br>$\mu_3 = 29695$, $\alpha_3 = -2.00$<br>D1 = D2 = D3 = 0	3D-Tetrahedron	

MEL = Model Extracted from Literature.

SSEL = Stress/Strain behavior Extracted from Literature, potentials fitted, to create a material model.

SM = Specific Measurements to create a material model.

Heel Pad and Achille Tendon materials properties

In vivo force-strain relationship of the Medial Gastrocnemius muscle (MG) was obtained using Ultrasonography (US) (to track the displacement of the muscle-tendon junction) and a custom ergometer (to record the muscle torque developed around the ankle joint). The subjects were asked to develop ramp-up contractions from rest to their maximal force. After processing the US images and torque data, the MG tendon force-strain relationship was modeled using a third-order Ogden hyperelastic model and used as mechanical characteristics of the Achilles tendon[29].

For the heel pad, mainly constituted of fat chambers, and used in compression during the impact of the foot on the ground, a specific material model, based on a first-order hyperelastic Ogden potential formulation, has been developed and validated using a FE sub-model. A set of X-ray radiography to track the deformation of the heel pad tissues and the displacement of the calcaneus for different loadings of the foot has been used as control data.

<u>Legend:</u>

Figure – Supplementary materials n°1: (**a**) View of the segmented surfaces extracted from CT scan data or reconstructed from anatomical data. Bones (grey), Achilles tendon (pink salmon), ankle's ligaments (yellow), ankle's cartilages (black), intrinsic muscles and plantar fascia (red), retinaculum (pink). Other parts, like the tendons or the tendons pulley, are directly constructed in the FE model, and not shown here. (**b**) Illustration of the meshing configuration for the bones (grey), heel pad (pink), ligaments (Yellow), cartilages (light blue), Achilles tendon (sky blue), tendons (dark blue), and tendon's pulley (red). For a sake of simplicity, only a part of the structures is represented.
